# Unexpected versus all-cause mortality as the endpoint for investigating the effects of a Rapid Response System in hospitalized patients

**DOI:** 10.1186/s13054-016-1339-9

**Published:** 2016-06-02

**Authors:** Anja H. Brunsveld-Reinders, Jeroen Ludikhuize, Marcel G. W. Dijkgraaf, M. Sesmu Arbous, Evert de Jonge, for the COMET study group

**Affiliations:** Department of Intensive Care, Leiden University Medical Center, PO Box 9600, Leiden, 2300 RC Netherlands; Department of Clinical Epidemiology, Leiden University Medical Center, Leiden, Netherlands; Department of Anesthesiology, Academic Medical Center, Amsterdam, Netherlands; Department of Clinical Research Unit, Academic Medical Center, Amsterdam, Netherlands

**Keywords:** Limitations of medical treatment, Patient safety, Unexpected death, Rapid response team, Medical record

## Abstract

**Background:**

The purpose of this study was to assess the effect of replacing all-cause mortality by death without limitation of medical treatments (LOMT) as the endpoint in a study of rapid response teams (RRTs) in hospitalized patients. We also described the time course of LOMT orders in patients dying on a general ward and the influence of RRTs on such orders.

**Methods:**

This study is a secondary analysis of the COMET trial, a pragmatic prospective Dutch multicenter before-after study. We repeated the original analysis of the influence of RRTs on death before hospital discharge by replacing all-cause mortality by death without an LOMT order. In a subgroup of all patients dying before hospital discharge, we documented patient demographics, admission characteristics and LOMT orders of each patient. Patients age 18 years or above were included.

**Results:**

In total, 166,569 patients were included in the study. The unadjusted ORs were 0.865 (95 % CI 0.77-0.98) in the original analysis using all-cause mortality and 0.557 (95 % CI 0.40-0.78) when choosing death without LOMT as the endpoint. In total, 3408 patients died before discharge. At time of death, 2910 (85 %) had an LOMT order. Median time from last change in LOMT status and death was 2 days (IQR 1–5) in the before-phase and median time after introduction of the RRT was 1 day (IQR 1–4) (*p* value not significant).

**Conclusions:**

The improvement in survival of hospitalized patients after introduction of a rapid response team in the COMET study was more pronounced when choosing death without limitation of medical treatment, rather than all deaths as the endpoint. Most patients who died during hospitalization had limitation of medical treatments ordered, often shortly before death. Rapid response teams did not influence the institution of limitation of medical treatments.

## Background

Patients who are admitted to general wards in hospitals may deteriorate, which may result in unplanned ICU admission, cardiac arrest, or even death [1]. Rapid response systems have been developed for timely identification and treatment of patients on general wards, who are at risk of clinical deterioration [2]. These systems have different names in the literature, including rapid response team, outreach team, or medical emergency team. In this paper we will use the term rapid response team (RRT) for both the actual outreach team and the rapid response system as a whole.

Three large controlled studies investigated the effects of the introduction of an RRT on clinical outcomes [3–5]. The endpoints of these studies were mortality, unplanned ICU admission and cardiac arrest rates. While studies in the UK and the Netherlands reported improved survival [4, 5] and decreased cardiac arrest rates [4], in an Australian study there was no improvement in a composite endpoint including mortality, unplanned ICU admission, and cardiac arrests [3].

Crude mortality may not be the optimal endpoint for studying the effects of an RRT on survival. Patients with untreatable diseases may be admitted to a hospital for palliative end-of-life care. Clearly, RRTs are not set up to prevent death in those patients. For this reason, unexpected death has been proposed as a more suitable endpoint for studying the effects of RRTs on survival [3]. Death was considered as expected if a patient was subject to a limitation of medical treatment (LOMT) order at the time of death. This, however, may not be the correct definition of expected death. First, some patients may prefer not to undergo life-sustaining treatments in the event of cardiac arrest, but this does not mean that death is imminent or that these patients do not want optimal treatment. Furthermore, treatment limitation orders are sometimes instituted shortly before death when the clinical condition has deteriorated progressively to a point that survival is no longer considered possible. Clearly, RRTs could have been beneficial in these patients if called in an earlier phase when the clinical condition was not yet hopeless.

The aim of our study was to explore the association between treatment-limitation orders and hospital death in a multicenter study of RRTs in the Netherlands. First, what is the effect of an RRT on mortality if all-cause hospital mortality is replaced by the endpoint of death without an LOMT order? Second, what proportion of patients dying on a general hospital ward are given an LOMT order, how do these LOMT orders change over time during hospitalization, and are LOMT policies influenced by the introduction of an RRT.

## Methods

### Design, setting, and participants

**Fig. 1:**
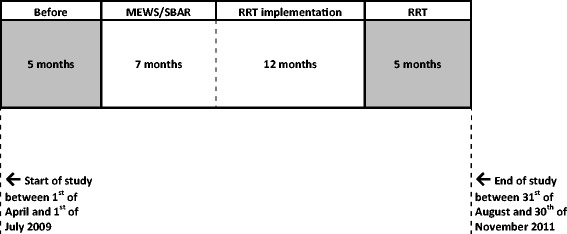
Design of the Cost and Outcomes analysis of Medical Emergency Teams (COMET) study. Following the baseline period of 5 months, the modified early warning score (MEWS)/situation background assessment recommendation (SBAR) was implemented for 7 months and subsequently followed up for 17 months during which the rapid response team (RRT) was available. Effects of the RRT on outcomes were measured during the last 5 months and compared with the 5 months baseline period. During the entire length of the study, data were collected on all the endpoints. For further clarification, hospitals were able to start with the study in a 3-month time period. The total study took 30 months, in which each hospital participated for 27 months

This study is a part of the Cost and Outcomes analysis of Medical Emergency Teams (COMET) multi-center study. The COMET study was designed as a prospective pragmatic before-after trial enabling the analysis of clinical outcomes after sequential introduction of the rapid response system components. Twelve Dutch hospitals participated in this study. Four study wards, comprising two surgical and two medical wards were included in each hospital, the so-called COMET wards. Patients included were 18 years of age or above. The full design of this study has been described previously [4, 6] and is shown in Fig. 1.

The study consisted of a before-period followed by two study phases. The before-period comprised 5 months during which baseline characteristics were collected. After that a two-step implementation of the RRT was performed. The first phase lasted 7 months during which the modified early warning score (MEWS) and the situation background assessment recommendation (SBAR) communication tools were implemented. The RRT was introduced during the second phase, which lasted 17 months. This phase was divided into the RRT implementation phase and the final RRT phase. The before-period and the final RRT phase were used to compare the effects on the outcome of the patients. To exclude seasonal effects on the outcome, the before-period and the final RRT phase in each hospital covered the same calendar months.

### Definitions

Unexpected death was defined as all deaths in patients without a pre-existing LOMT order [3, 7]. Definitions of the LOMT in this study were: Code A for “full active care”, Code C for “do not perform cardiopulmonary resuscitation” and/or “do not admit to ICU”, and Code D for “only palliative care”. Code B had been used in the past, but was no longer used in any of the participating hospitals. In this study, if no LOMT was recorded in the charts, this was considered equivalent to code A “for full active care”.

### Ethical consideration

The medical ethics committee of the Academic Medical Center in Amsterdam waived the need for formal evaluation of the study due to the observational nature of the study. Consequently, the need for informed consent was not applicable.

### Intervention

All deaths were recorded during the study period using a clinical report form. All deaths included those in patients who had been admitted to the COMET ward and transferred at a certain point to a non-COMET ward. Clinical information systems in the hospitals were used to identify death during this study. We collected the following data: basic patient demographic data (age and gender), admission characteristics (date of admission, date of transfer to the COMET ward, COMET ward specialty, length of hospital stay, and date and time of death), and limitation of medical treatment (date of recorded LOMT).

After implementation of the RRT, members of the RRT collected the following data during consultation: the personnel who activated the RRT, the indication for summoning the RRT, the direct outcome after RRT, and the treatment code before and after consultation.

### Statistical analysis

Data analysis was performed using SPSS version 20.0 (Armonk, New York, USA). Generalized linear mixed modeling (GLMM) was applied to assess differences in outcomes per 1000 admissions between the before-period and final RRT period, while correcting for potential confounding due to the before-after study design. Death was assumed to have a binominal distribution in the GLMM. Potential confounders were included as fixed or random variables. Hospital was modeled as a random variable. The age of patients was modeled as a random component, whereas patient’s sex and admission type (planned vs unplanned/emergency) were modeled as fixed variables. The uncorrected odds ratios (ORs) and ORs after correction for confounding are reported with their CIs and corresponding *p* values. Descriptive analyses are presented as raw numbers and percentages. Continuous data are presented as medians with interquartile range (IQR) due to the non-normally distributed data. The non-parametric Mann–Whitney *U* test was used to compare non-normally distributed continuous variables between groups. Categorical variables were compared between groups using the chi squared (χ^2^) test. The level of significance was set at *p* < 0.05.

## Results

In total 166,569 patients were included in the COMET study, of whom 2345 patients died on a medical ward and 1063 patients on a surgical ward. Of the patients who died, surgical patients were older median 81.4 years (IQR 73.6–87.0) in comparison to medical patients, median 78.4 years (68.3–85.6). The median hospital length of stay (LOS) was 7 days (IQR 3–16 days) for surgical patients compared to 6 days (3 to 13 days) for medical patients. In 13 % of patients who died and for whom an RRT was called, an LOMT was instituted or changed after consultation of the RRT. Baseline characteristics of patients are presented in Table 1.

**Table 1:** Demographic data

		Medical	Surgical
Deaths, *n*		2345	1063
Implementation phases of the rapid response system, *n* (%)	Before	387 (17)	189 (18)
	MEWS	643 (27)	267 (25)
	RRT implementation	940 (40)	460 (43)
	Final RRT	375 (16)	147 (14)
Gender, male, *n* (%)		1261 (54)	1084 (54)
Age, years, median (IQR)		78.4 (68.3–85.6)	81.4 (73.6–87.0)
Death on Intensive Care Unit, *n* (%)		48 (2)	43 (4)
Time of death, *n* (%)	0000–0559 h	701 (30)	302 (28)
	0600–1159 h	555 (24)	255 (24)
	1200–1759 h	530 (23)	245 (23)
	1800–2359 h	508 (22)	241 (23)
	Unknown	51 (2)	20 (2)
Hospital length of stay, median (IQR)		6 (3–13)	7 (3–16)
Number of RRT consultations before death		56 (45)	68 (55)
	0–24 h	45 (80)	62 (92)
	24–48 h	3 (5)	5 (7)
	>48 h	8 (14)	1 (1)
Initiation of LOMT order by RRT		7 (13)	9 (13)

*RRT* rapid response team, *LOMT* limitation of medical treatment

The ORs for death before hospital discharge for patients admitted during the last 5 months of the RRT phase (n = 27,820) were compared with the baseline period before implementing the RRT (n = 26,659). The originally reported unadjusted OR for all-cause mortality in the final RRT period compared to the before period was 0.865 (95 % CI 0.77-0.97) [4]. In the same cohort of patients, the unadjusted OR for death without LOMT (unexpected death) was 0.557 (95 % CI 0.40-0.78). Likewise, the ORs after adjustment for age, gender, individual hospital, and urgent vs planned admission were 0.802 (95 % CI 0.64-1.0) in the original analysis using all-cause mortality and 0.549 (95 % CI 0.38-0.78) when choosing death without LOMT as the endpoint (Table 2).

**Table 2:** Comparison of effect of RRT on all-cause in-hospital mortality vs death without LOMT in hospitalized patients

	Uncorrected OR	95 % CI of uncorrected OR	Corrected OR	95 % CI of corrected OR	*P* value for corrected OR
Deaths, *n*/1000 (95 % CI)	0.865	0.768-0.975	0.802	0.644-1.0	0.05
Death without LOMT, *n*/1000 (95 % CI)	0.557	0.397-0.782	0.549	0.385-0.784	0.001

Odds ratios (OR) represent differences between final rapid response team (RRT) phase vs the before-phase. Corrected ORs are adjusted for sex, age, hospital, and urgency of admissions. Number of admissions in the before-period = 26,659; number of admissions in the RRT period = 27,820. *LOMT* limitation of medical treatment

Table 3 shows the treatment limitations at different time points in patients who died during hospital admission. In both medical and surgical patients, most of the patients who subsequently died already had an LOMT at hospital admission. The median time between last LOMT order and death was 3 days in patients who were assigned Code C and 1 day in patients assigned code D. There was also a short time between the issue of the LOMT order and death in patients who had a prolonged hospital length of stay. Unexpected death was defined as death without a pre-existing LOMT order. There was no LOMT order at the time of death in 12 % of medical patients and 20 % of surgical patients.

**Table 3:** Limitation of medical treatment (LOMT) order status at different time points in patients who died during hospital admission

		Medical		Surgical	
		*n* (%)	Days^a^	*n* (%)	Days^a^
	All deaths	2345	2 (1–5)	1063	1 (1–5)
LOMT at time of admission	Code A	736 (31)		459 (43)	
	Code C	1278 (55)		464 (44)	
	Code D	331 (14)		140 (13)	
LOMT at time of death	Code A	280 (12)	5 (1–10)	218 (21)	4 (1–11)
	Code C	790 (34)	3 (1–8)	352 (33)	3 (1–8)
	Code D	1275 (54)	1 (0–2)	493 (46)	1 (0–2)
Change in LOMT status between admission and death	Code A–A	279 (12)		217 (20)	
	Code A–C	137 (6)	3 (1–8)	79 (7)	3 (0–7)
	Code A–D	320 (14)	1 (0–2)	163 (15)	1 (0–2)
	Code C–C	649 (28)		273 (26)	
	Code C–D	629 (27)	1 (0–2)	190 (18)	1 (0–2)
	Code C–A	0 (0)	NA	1 (0)	0
	Code D–D	326 (14)		140 (13)	
	Code D–C	4 (0)	5 (2–30)	0 (0)	NA
	Code D–A	1 (0)	8	0 (0)	NA
Length of hospital stay	0–3 days	762 (32)	1 (0–2)	324 (30)	1 (0–2)
	4–7 days	541 (23)	2 (1–5)	228 (21)	3 (1–5)
	8–14 days	517 (22)	3 (1–9)	217 (20)	2 (1–9)
	15–21 days	219 (9)	3 (1–12)	101 (10)	2 (1–15)
	>21 days	306 (13)	3 (1–20)	193 (18)	3 (1–26)

Data are presented as number (%) or median (IQR). ^a^Delta time between last code change and time of death. *Code A* patients who were to have full active care, *Code C* patients who were not to have cardiopulmonary resuscitation and/or were not to be admitted to ICU, *Code D* patients who were to have only palliative care

In Table 4 the effect of RRT implementation on treatment limitations in patients who died during their hospital stay is presented. No differences were found in institution of LOMT after introduction of the rapid response system. The delta time between the last code change and death was 2 days (median 1–5) in the before-phase and 1 day (median 1–4) in the final RRT phase; this was not significant.

**Table 4:** Effects of implementation of rapid response system on limitation of medical treatment (LOMT) order status

		Before	Final RRT	
		(n = 576)	(n = 522)	*P* value*
LOMT at time of admission, *n* (%)	Code A	221 (38)	187 (36)	0.31
	Code C	271 (47)	269 (52)	
	Code D	84 (15)	66 (13)	
LOMT at time of death, *n* (%)	Code A	99 (17)	64 (12)	0.06
	Code C	170 (30)	174 (33)	
	Code D	307 (53)	284 (54)	
Delta time, days, between last change in LOMT status and death, median (IQR)		2 (1–5)	1 (1–4)	0.09
Stratified by hospital length of stay, median (IQR) (*n*)	0–3 days	1 (0–2) (195)	1 (0–2) (178)	0.74
	4–7 days	3 (1–5) (130)	2 (1–5) (110)	0.27
	8–14 days	3 (1–9) (100)	2 (1–7) (125)	0.09
	15–21 days	2 (1–10) (54)	3 (1–15) (38)	0.55
	>21 days	5 (1–25) (97)	2 (1–12) (71)	0.12

Medical and surgical patients are combined. *Code A* patients who were to have full active care, *Code C* patients who were not to have cardiopulmonary resuscitation and/or were not to be admitted to ICU, *Code D* patients who were to have only palliative care, *RRT* rapid response team. *Chi-square or Mann–Whitney *U* test was used as appropriate

## Discussion

In this study we demonstrate that the effects of introducing an RRT on death in hospital is more pronounced if death without LOMT is used as the endpoint compared to all-cause mortality as was used in the original COMET analysis [4]. The underlying hypothesis as to why death without LOMT might be a better endpoint than all deaths is that patients with LOMT are expected to die and for these patients an RRT call will not be initiated. Thus, it has been argued that the true effects of an RRT are underestimated if all patients are analyzed as was done in the original analyses in the COMET study [6]. In one earlier controlled trial on the effects of an RRT in Australian hospitals, unexpected death, i.e., death in patients with no LOMT, was included in the composite endpoint consisting of unplanned ICU admission, or cardiac arrest, or unexpected death. However, the negative findings in this study may be related to factors such as insufficient statistical power and contamination of the control group [3, 8, 9].

In this cohort of patients who all died before hospital discharge, 85 % had some LOMT at the end of life. At hospital admission 65 % of patients who died in hospital had LOMT. We are not the first to show that most hospitalized patients who eventually die have LOMT. In a study from Canada and the USA, in a cohort of patients with community-acquired pneumonia who required admission to a hospital, 51 of 65 patients (78 %) who died had do-not-resuscitate orders instituted before death [10]. In 1995 in the USA, among a representative sample of Medicare patients hospitalized with congestive heart failure, acute myocardial infarction, pneumonia, cerebrovascular accident, or hip fracture, 49 % of patients who died had LOMT orders [11]. In a study in Saudi Arabia, after implementing an RRT, 2793 out of 3191 patients (88 %) dying in hospital, died on the general ward with LOMT orders instituted [12].

Patients with an LOMT are believed not to benefit from the RRT because death is expected. This, however, is not necessarily true. First, there may be many reasons for limiting medical treatments. Patients may prefer not to undergo some invasive procedures, such as mechanical ventilation, or physicians may consider treatments inappropriate due to a patient’s poor prognosis. In both circumstances, patients may still be successfully treated and discharged from the hospital. Moreover, in our study, we found that 84 % of patients who died had some limitation of medical treatment at the time of death. However, in most of these patients the LOMT order was instituted in the last days before death, sometimes even less than one day earlier. Thus, having treatment limitations at the time of death cannot be interpreted as death being expected during the entire hospital stay. It appears that LOMT instituted shortly before death is more a reflection of the deteriorating condition of the patient during their hospital stay, eventually leading to the clinical conclusion that death is inevitable and that some treatments would be better withheld. It does not imply that RRT could not have improved the outcome in the earlier period in these patients.

RRTs have been installed in hospitals with the aim of timely identification and treatment of patients deteriorating on general wards, and preventing morbid outcomes. An additional role of the RRT is to be involved in decisions and discussions with the physicians on the ward about palliative care and LOMT if patients have no real prospect of surviving with a reasonable quality of life [13]. In an earlier study, an RRT was associated with improved documentation of comfort care orders, pain scores, patient distress, and chaplain visits [14]. In a recent review, Jones and coworkers mentioned several reasons why RRTs may need to be involved in end-of-life decisions. First, the usual care team may not have recognized or may not accept that the patient is dying. Second, the usual team may not be comfortable or skilled in having end-of-life care discussions with patients or families. Last, the usual team may have difficulty in accepting LOMT despite the presence of advanced comorbidities and an irreversible new illness, due to personal or religious reasons [15]. Also, RRTs may confront situations in which LOMT orders are postponed awaiting discussion with team or family members [16].

In our study 13 % of RRT calls were followed by the institution of LOMT orders. This is less than found by others. Smith and coworkers reported that 28 % of RRT activations were associated with new LOMT orders [17]. Casamento and coworkers reported LOMT orders after 32 % of RRT calls [18]. In a study by Jones et al., 31 % of RRT activations were associated with LOMT [19]. A possible explanation for the low rate of LOMT orders after RRT calls in our study is the already high prevalence of LOMT orders at hospital admission. It appears that most patients at the end of life already had LOMT before the RRT was called. Accordingly, in our study, we found no differences in the institution of LOMT before and after implementation of the RRT, although the relatively small number of patients cannot exclude a small effect in favor of the RRT period.

In this study there are some limitations. First, during the review of the medical charts of the patients who died, we assumed that medical treatments were not limited if there was no LOMT order recorded in the patient charts. However, it is possible that implicit limitations of medical treatment were present in some of these cases. Therefore, we cannot exclude some underestimation of the LOMT during this study and consequently an overestimation of the number of patients dying unexpectedly. Second, to estimate the effect of replacing all-cause hospital mortality by death without LOMT when studying the effects of the RRT, patients dying with LOMT were considered as not having reached the endpoint, but as patients surviving up to hospital discharge. Preferentially, patients with LOMT orders should be excluded from the study population. However, as information about LOMT was only present for patients who died, this was not possible. When excluding only patients who died with LOMT, we found ORs that were almost identical to those presented here. As relatively few patients surviving up to hospital discharge have LOMT orders, we believe that it is unlikely that these patients have major influence on our findings. Our study was performed in 12 hospitals, thereby increasing the generalizability of our findings. However, all centers were in the Netherlands and it important to realize that common policies about treatment limitations differ between countries [20]. Last, we had a relatively low percentage of RRT calls recorded during this study. This may be due to administrative concerns. It was not always clear to the physician of the ward when to call the RRT or to call the ICU for rapid consultation. Thus, the real number of RRT calls may have been higher than documented.

## Conclusions

We observed improved survival up to hospital discharge when choosing the endpoint of death without LOMT rather than all deaths, in a study on the effect of implementation of RRTs in Dutch hospitals. Implementation of rapid response systems was not associated with significant change in LOMT. Most patients who died during hospitalization had LOMT orders instituted, often shortly before death. LOMT does not necessarily mean that death is expected and that these patients could not benefit from treatment by the rapid response team.

## Key messages

- Replacing all-cause mortality by death without LOMT (unexpected death) results in improved survival up to hospital discharge
- Most patients who died during hospitalization had LOMT orders instituted, often shortly before death
- The presence of LOMT does not necessarily mean that death is expected and that these patients could not benefit from treatment by the rapid response team

## Abbreviations

CI: confidence interval
COMET: Cost and outcomes analysis of medical emergency teams
GLMM: generalized linear mixed modeling
ICU: Intensive Care Unit
IQR: interquartile range
LOMT: limitation of medical treatments
LOS: length of stay
MEWS: modified early warning score
OR: odds ratio
RRT: rapid response teams
SBAR: situation background assessment recommendation
